# The Impact of Leadered and Leaderless Gene Structures on Translation Efficiency, Transcript Stability, and Predicted Transcription Rates in Mycobacterium smegmatis

**DOI:** 10.1128/JB.00746-19

**Published:** 2020-04-09

**Authors:** Tien G. Nguyen, Diego A. Vargas-Blanco, Louis A. Roberts, Scarlet S. Shell

**Affiliations:** a Department of Biology and Biotechnology, Worcester Polytechnic Institute, Worcester, Massachusetts, USA; b Program in Bioinformatics and Computational Biology, Worcester Polytechnic Institute, Worcester, Massachusetts, USA; Ohio State University

**Keywords:** 5′ UTR, leaderless translation, *Mycobacterium tuberculosis*, mRNA stability, posttranscriptional control mechanisms, sigma factors, smegmatis, transcription

## Abstract

Regulation of gene expression is critical for Mycobacterium tuberculosis to tolerate stressors encountered during infection and for nonpathogenic mycobacteria such as Mycobacterium smegmatis to survive environmental stressors. Unlike better-studied models, mycobacteria express ∼14% of their genes as leaderless transcripts. However, the impacts of leaderless transcript structures on mRNA half-life and translation efficiency in mycobacteria have not been directly tested. For leadered transcripts, the contributions of 5′ untranslated regions (UTRs) to mRNA half-life and translation efficiency are similarly unknown. In M. tuberculosis and M. smegmatis, the essential sigma factor, SigA, is encoded by a transcript with a relatively short half-life. We hypothesized that the long 5′ UTR of *sigA* causes this instability. To test this, we constructed fluorescence reporters and measured protein abundance, mRNA abundance, and mRNA half-life and calculated relative transcript production rates. The *sigA* 5′ UTR conferred an increased transcript production rate, shorter mRNA half-life, and decreased apparent translation rate compared to a synthetic 5′ UTR commonly used in mycobacterial expression plasmids. Leaderless transcripts appeared to be translated with similar efficiency as those with the *sigA* 5′ UTR but had lower predicted transcript production rates. A global comparison of M. tuberculosis mRNA and protein abundances failed to reveal systematic differences in protein/mRNA ratios for leadered and leaderless transcripts, suggesting that variability in translation efficiency is largely driven by factors other than leader status. Our data are also discussed in light of an alternative model that leads to different conclusions and suggests leaderless transcripts may indeed be translated less efficiently.

**IMPORTANCE** Tuberculosis, caused by Mycobacterium tuberculosis, is a major public health problem killing 1.5 million people globally each year. During infection, M. tuberculosis must alter its gene expression patterns to adapt to the stress conditions it encounters. Understanding how M. tuberculosis regulates gene expression may provide clues for ways to interfere with the bacterium’s survival. Gene expression encompasses transcription, mRNA degradation, and translation. Here, we used Mycobacterium smegmatis as a model organism to study how 5′ untranslated regions affect these three facets of gene expression in multiple ways. We furthermore provide insight into the expression of leaderless mRNAs, which lack 5′ untranslated regions and are unusually prevalent in mycobacteria.

## Abstract (precis)

Tuberculosis, caused by Mycobacterium tuberculosis, is a major public health problem killing 1.5 million people globally each year. During infection, M. tuberculosis must alter its gene expression patterns to adapt to the stress conditions it encounters. Understanding how M. tuberculosis regulates gene expression may provide clues for ways to interfere with the bacterium’s survival. Gene expression encompasses transcription, mRNA degradation, and translation. Here, we used Mycobacterium smegmatis as a model organism to study how 5′ untranslated regions affect these three facets of gene expression in multiple ways. We furthermore provide insight into the expression of leaderless mRNAs, which lack 5′ untranslated regions and are unusually prevalent in mycobacteria.

## INTRODUCTION

The pathogen Mycobacterium tuberculosis has evolved numerous strategies to survive in different niches within the human host. Bacterial adaptation to these harsh environments is usually achieved by gene regulation, both transcriptionally and posttranscriptionally. While promoters play critical roles in gene regulation, other gene features and mechanisms have additional important regulatory roles. One such important gene feature is the 5′ untranslated region (5′ UTR), which contains the Shine-Dalgarno (SD) sequence within the ribosome binding site (RBS) and, therefore, can serve as a translation regulator (1–5). For example, 5′ UTR interactions with *cis* and *trans* elements, such as complementary sequences within the UTR or coding sequence, small RNAs (sRNAs), and RNA-binding proteins, can modulate protein synthesis by blocking or improving accessibility to the RBS (6–9). Importantly, it has been shown in Escherichia coli and other bacteria that transcription and translation are physically coupled, and thus 5′ UTR-mediated modulation of translation could have repercussions on transcription rate as well (10–14). Translation blocks in Mycobacterium smegmatis have been shown to decrease transcription as well (15), suggesting that transcription-translation coupling occurs in mycobacteria, although the extent and consequences are unknown.

The 5′ UTRs can also regulate gene expression by altering mRNA turnover rates. This can be a consequence of altered translation rates, as impairments to translation often lead to faster mRNA decay (16–22). In other cases, mRNA stability is directly affected by sRNA binding to 5′ UTRs or by UTR secondary structure (9, 23–28). In E. coli, the half-life of the short-lived transcript *bla* can be significantly increased when its native 5′ UTR is replaced with the 5′ UTR of *ompA*, a long-lived transcript (29–31). Conversely, deletion of *ompA*’s native 5′ UTR decreased its half-life by 5-fold (30). The longevity conferred by the *ompA* 5′ UTR was attributed to the presence of a nonspecific stem-loop as well as the specific RBS sequence (30–32). Secondary structure formation in 5′ UTRs has been shown to play a major role in transcript stability in other bacteria as well, such as for *ermC* in Bacillus subtilis (33, 34) and *pufBA* in Rhodobacter capsulatus (35–37). Moreover, obstacles that hinder the linear 5′ scanning function of RNase E (a major RNase in E. coli and mycobacteria) can prevent access to downstream cleavage sites, increasing transcript half-life (38). Such obstacles include the 30S ribosomal subunit bound to an SD-like site far upstream of the translation start site in one case (39). UTRs can also contain binding sites for the global regulator CsrA, which can both promote and prevent mRNA decay in E. coli (40). Although effects of 5′ UTRs on mRNA stability, translation, and transcription rate have been widely studied in more common bacterial systems, there is a paucity of information of the regulatory effects of 5′ UTRs in mycobacteria.

Compared to E. coli and most other well-studied bacteria, mycobacteria possess a large number of leaderless transcripts; approximately 14% of annotated genes are leaderless in both M. smegmatis and M. tuberculosis (41–43). Studies in E. coli have shown that translation of leadered and leaderless transcripts is functionally distinct (44–50), suggesting fundamental differences in their mechanisms of regulation. In contrast to E. coli, where leaderless transcripts are generally translated less efficiently (42, 51–54), leaderless transcripts in mycobacteria appear to be translated robustly (42, 43). However, direct comparisons of translation rates for leadered versus leaderless transcripts in mycobacteria have yet to be reported.

Among leadered transcripts, 5′ UTR lengths vary. We hypothesized that longer 5′ UTRs were more likely to play regulatory roles through modulation of translation, transcription rate, and mRNA turnover. One such long-leadered transcript in both M. tuberculosis and M. smegmatis encodes sigma factor alpha (*sigA*), the primary sigma factor in mycobacteria (55, 56). Here, we used the mycobacterial model M. smegmatis and a series of yellow fluorescent protein (YFP) reporters to investigate the effects of the *sigA* 5′ UTR as well as leaderless gene structures on transcription, translation, and mRNA half-life. We found that the *sigA* 5′ UTR caused lower translation efficiency, reduced mRNA half-life, and a higher predicted transcript production rate compared to those of a control 5′ UTR. Leaderless transcripts were translated at similar rates as those of transcripts bearing the *sigA* 5′ UTR and had similar half-lives but appeared to be transcribed less efficiently, leading to lower steady-state mRNA and protein abundances. Our results highlight the potential of 5′ UTRs to affect transcription efficiency as well as translation and mRNA half-life and support the idea that leaderless translation can be either more or less efficient than leadered translation in mycobacteria, depending on the characteristics of the leader. Alternative interpretations of our data are possible and would lead to different conclusions, particularly with respect to the efficiency of leaderless translation. These will be discussed.

## RESULTS

### Validation of the *sigA* 5′ UTR boundaries.

**FIG 1:**
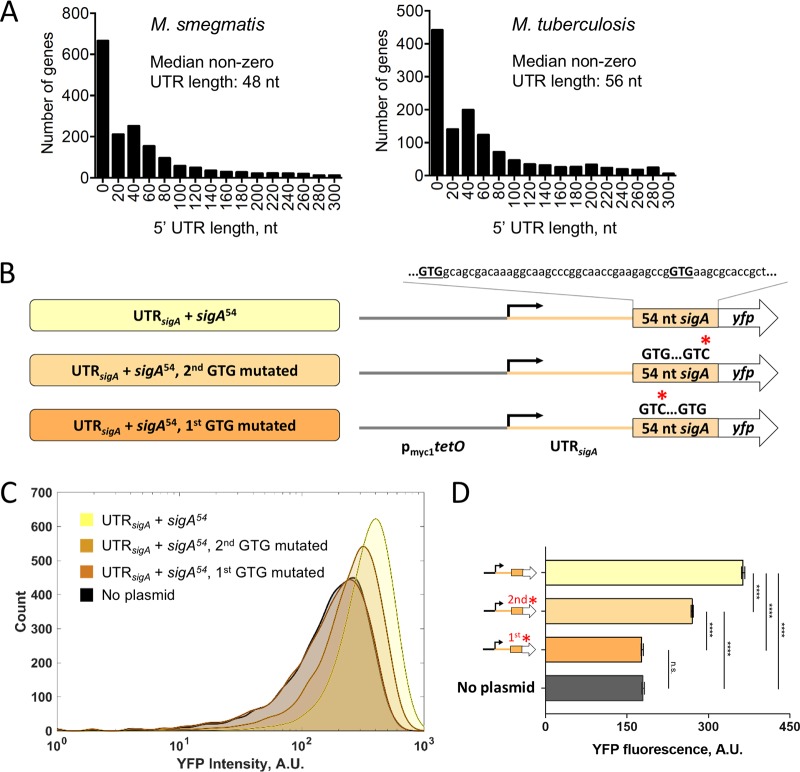
The M. smegmatis *sigA* gene has a longer-than-typical 5′ UTR. (A) Distributions of 5′ UTR lengths for M. smegmatis and M. tuberculosis genes reported to be transcribed from a single TSS (41, 42). (B) Constructs to confirm the predicted *sigA* translation start site. p_myc1_*tetO* was described in reference 57. UTR*_sigA_* denotes the 123-nt sequence between the experimentally determined TSS (41) and the annotated translation start site. (C) Flow cytometry with YFP-expressing constructs diagrammed in panel B. (D) Median fluorescence intensities determined by flow cytometry. Error bars denote 95% confidence interval (CI). Fluorescence intensities were compared by Kruskal-Wallis test followed by Dunn’s multiple-comparison test. ****, *P* < 0.0001; ns, *P* > 0.05.

Transcription start site mapping has defined the 5′ ends of 5′ UTRs on a transcriptome-wide basis in both M. smegmatis and M. tuberculosis (41, 42). Using annotated translation start sites to define the 3′ ends of the 5′ UTRs, the median 5′ UTR lengths in M. smegmatis and M. tuberculosis are 48 and 56 nucleotides (nt), respectively, after excluding leaderless genes (Fig. 1A; see also Table S1 in the supplemental material) (41, 42). The 5′ UTR length distributions are skewed, with a mode of approximately 40 nt (Fig. 1A). We hypothesized that longer-than-average 5′ UTRs are more likely to have regulatory roles and sought to investigate the role of the 5′ UTR of the M. smegmatis *sigA* gene. The M. tuberculosis *sigA* 5′ UTR (128 nt) is also predicted to be longer than the median. To ensure that the predicted 5′ UTR boundaries of M. smegmatis *sigA* were correct, we experimentally validated the predicted start codon at genome coordinate 2827625 in GenBank accession number NC_008596, which resulted in a 123-nt UTR. A second GTG codon 39 nt downstream at 2827586 also had an appropriately positioned Shine-Dalgarno-like sequence and could conceivably be used as a start codon. We therefore made reporter constructs in which the strong constitutive promoter p_myc1_*tetO* (57) drove expression of a transcript containing the *sigA* 5′ UTR and the sequence encoding YFP, with a C-terminal 6×His tag and an N-terminal fusion of the sequence encoded by the first 54 nt of the annotated *sigA* coding sequence. We then individually mutated each of the two putative GTG start codons to GTC (Fig. 1B). Mutations of the first GTG to GTC reduced fluorescence to levels indistinguishable from autofluorescence in a strain that lacked the *yfp* gene (Fig. 1C and D, no plasmid). In contrast, mutation of the second GTG to GTC reduced fluorescence to an intermediate level (Fig. 1C and D). We therefore concluded that the first GTG is likely to be the predominant site of translation initiation, while the second GTG may affect expression levels but is not by itself sufficient to produce above-background expression. For subsequent experiments, we considered the first GTG to be the most likely start of the coding sequence and thereby define the *sigA* 5′ UTR as 123 nt in length.

### Assumptions made in subsequent data analysis.

In subsequent sections, we will report data on mRNA abundance, mRNA half-life, and protein abundance for a series of reporter constructs. We will also report predicted transcription rates and apparent translation efficiencies, which are calculated from the abundance and half-life data. These calculated values rest upon a key assumption that most of the mRNA synthesized in the cell contributes to the measured abundance and half-life values. If this is not true, the data could be interpreted differently and different conclusions reached (58). These alternate interpretations are offered in Discussion.

### The initial portion of the *sigA* coding sequence affects mRNA half-life and predicted transcript production rate.

**FIG 2:**
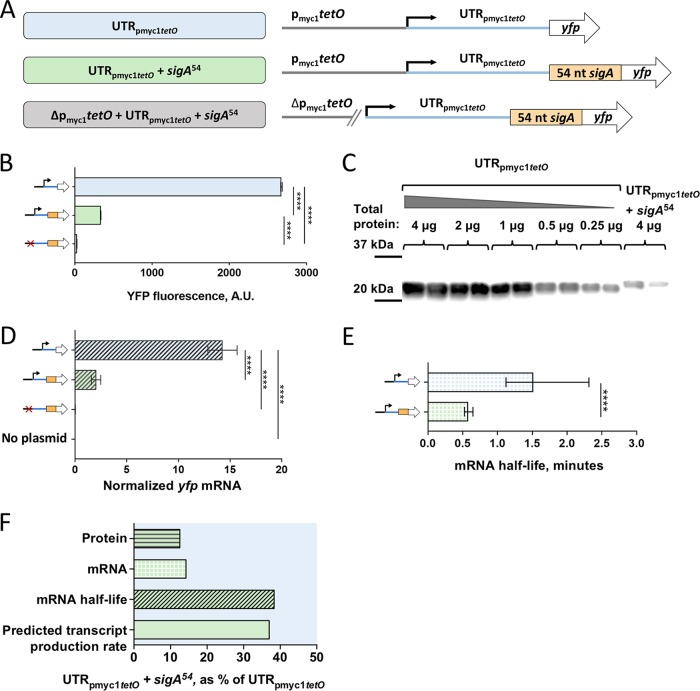
The first 54 nt of the *sigA* coding sequence affects transcript production rate and mRNA half-life. (A) Constructs transformed into M. smegmatis to determine the impact of the first 54 nt of the *sigA* coding sequence (*sigA*^54^) on expression of a YFP reporter. (B) Median YFP fluorescence of strains bearing the constructs in panel A, determined by flow cytometry. Error bars denote 95% CI. Strains were compared by Kruskal-Wallis test followed by Dunn’s multiple-comparison test. (C) Lysates from strains bearing constructs with and without *sigA*^54^ were subject to Western blotting to detect the C-terminal 6×His tag on the YFP. The mass of total protein loaded per lane is stated. (D) *yfp* mRNA abundance for strains bearing the indicated constructs, determined by qPCR and normalized to expression of endogenous *sigA*. Error bars denote standard deviation. Strains were compared by analysis of variance (ANOVA) with Tukey’s honestly significant difference (HSD) test. (E) The half-lives of *yfp* mRNA produced from the indicated constructs were measured. Error bars denote 95% CI. Half-lives were compared using linear regression analysis (*n* = 3). (F) Protein abundance, mRNA abundance, mRNA half-life, and calculated transcript production rate for the construct containing *sigA*^54^ are shown as a percentage of the values produced by a construct that lacks *sigA*^54^ but is otherwise identical. ****, *P* < 0.0001.

To capture 5′ UTR-dependent effects on transcription, mRNA stability, and translation, we sought to investigate the role of the *sigA* 5′ UTR (UTR*_sigA_*) in the context of a *yfp* transcript. UTR-mediated regulation of translation sometimes involves base pairing of 5′ UTR sequences with elements in the early portion of the coding sequence. Thus, we decided to include in our investigation the first 54 nt from the coding region of *sigA* (*sigA*^54^). To determine if *sigA*^54^ alone affected expression, we compared fluorescence from our YFP reporters with or without the *sigA*^54^ N-terminal extension, independent from UTR*_sigA_*. Transcription was driven by the p_myc1_*tetO* promoter for these and all constructs used in this study. While this semisynthetic promoter contains TetR binding sites, the strains used in this study did not encode the corresponding Tet repressor, and the promoter was therefore constitutively active. Where indicated, constructs included the p_myc1_*tetO*-associated 5′ UTR (UTR_pmyc1_*_tetO_*) as initially described in reference 57. To ensure that expression initiated only from the annotated promoter and not from spurious promoter-like sequences in UTR_pmyc1_*_tetO_* or *sigA*^54^, we built a control strain in which nt −53 through −1 of the promoter were deleted (Δp_myc1_*tetO*) (Fig. 2A).

We first tested the impact of *sigA*^54^ on YFP fluorescence intensity using UTR_pmyc1_*_tetO_*. Interestingly, the *sigA*^54^ strain was ∼9-fold less fluorescent than the strain in which YFP lacked this N-terminal extension (Fig. 2B). To confirm that the reduced YFP fluorescence in the presence of *sigA*^54^ indeed reflected reduced protein levels rather than altered YFP structure or intrinsic fluorescence, we measured protein levels directly by Western blotting. The Western blotting data were consistent with the flow cytometry result, showing an approximately 16-fold reduction of YFP levels with the inclusion of *sigA*^54^ compared to the no-*sigA*^54^ strain (Fig. 2C; see also Fig. S1 in the supplemental material).

To assess if the presence of *sigA*^54^ affected *yfp* transcript levels, we conducted quantitative PCR (qPCR) for the same set of strains. Indeed, *sigA*^54^ *yfp* levels were approximately 6-fold lower than those of the *yfp* strain (Fig. 2D). This suggested that the decrease in YFP protein levels could be due to a reduction in *yfp* mRNA levels. Alternatively, the *sigA*^54^ *yfp* transcript could be translated less efficiently, leading to reduced mRNA stability and thus lower steady-state abundance.

We then wondered if *sigA*^54^ affected transcript abundance by increasing the rate of transcript decay or by decreasing the rate of transcription. Thus, we determined mRNA half-life for *yfp* with and without *sigA*^54^. As shown in Fig. 2E, we estimated the half-life of *yfp* alone to be ∼1.5 min and the half-life of *yfp* plus *sigA*^54^ to be ∼0.6 min. We concluded that the first 54 nt of *sigA* made the *yfp* transcript more susceptible to degradation. Knowing the abundance and decay rate of a transcript, the rate of transcription can be predicted mathematically (59). This predicted transcription rate encompasses initiation, elongation, and termination, and changes in the apparent transcription rate could therefore theoretically result from changes in any of those three facets. We will henceforth refer to this calculated rate as the predicted transcript production rate. The insertion of *sigA*^54^ as an N-terminal extension for YFP appeared to reduce the *yfp* transcript production rate by approximately 60% (Fig. 2F).

### The *sigA* 5′ UTR affects transcript half-life, translation, and predicted transcript production rate.

**FIG 3:**
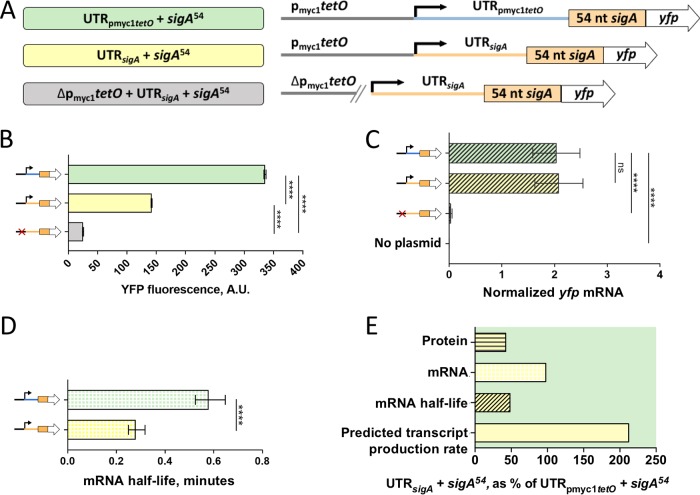
The *sigA* 5′ UTR affects translation efficiency, mRNA half-life, and transcript production rate. (A) Constructs transformed into M. smegmatis to determine the impact of the *sigA* 5′ UTR on expression of a YFP reporter. (B) Median YFP fluorescence of strains bearing the constructs in panel A, determined by flow cytometry. Error bars denote 95% CI. Strains were compared by Kruskal-Wallis test followed by Dunn’s multiple-comparison test. (C) *yfp* mRNA abundance for strains bearing the indicated constructs, determined by qPCR and normalized to expression of endogenous *sigA*. Error bars denote standard deviation. Strains were compared by ANOVA with Tukey’s HSD. (D) The half-lives of *yfp* mRNA produced from the indicated constructs were measured. Error bars denote 95% CI. Half-lives were compared using linear regression analysis (*n* = 3). (E) Protein abundance, mRNA abundance, mRNA half-life, and calculated transcript production rate for the construct containing the *sigA* 5′ UTR are shown as a percentage of the values produced by a construct that contains the p_myc1_*tetO*-associated 5′ UTR. Note that some data shown in Fig. 2 are reproduced here to facilitate comparisons. ****, *P* < 0.0001; ns, *P* > 0.05.

In order to assess the effects of UTR*_sigA_* on transcription, mRNA stability, and translation, we replaced UTR_pmyc1_*_tetO_* with UTR*_sigA_* in our *sigA*^54^ *yfp* reporters as shown in Fig. 3A. The presence of UTR*_sigA_* led to an approximately 2-fold reduction in YFP fluorescence intensity when compared to the UTR_pmyc1_*_tetO_* reporter strain (Fig. 3B). We wondered if the reduction in fluorescence attributed to UTR*_sigA_* was associated with reduced *yfp* transcript abundance. However, qPCR revealed equivalent transcript levels for strains with UTR*_sigA_* and UTR_pmyc1_*_tetO_* (Fig. 3C), indicating that the reduced protein levels were more likely a consequence of reduced translation efficiency. Interestingly, *yfp* mRNA half-life was reduced to 0.28 min by the presence of UTR*_sigA_* (Fig. 3D), suggesting that a higher transcript production rate is required to maintain the steady-state mRNA abundance that we observed (Fig. 3E). Taken together, our findings suggest that UTR*_sigA_* may affect transcription, transcript decay, and translation. In Fig. 3E, we summarize these results as percentages of *yfp* transcript production rate, mRNA abundance, mRNA half-life, and YFP protein levels relative to the UTR_pmyc1_*_tetO_ sigA*^54^ strain.

We analyzed the sequences and predicted secondary structures of UTR*_sigA_* and UTR_pmyc1_*_tetO_* to investigate possible causes of the apparent difference in translation efficiency. The ribosome binding sites (RBSs) of these two UTRs have similar degrees of identity to a theoretically perfect mycobacterial SD sequence (the reverse complement of the 3′ end of the 16S rRNA) (see Fig. S2A in the supplemental material). We noted that the spacing between the SD and start codon differed between the two UTRs (see Fig. S2A). However, both spacings are common among native M. smegmatis transcripts harboring these SD sequences (Fig. S2B), suggesting that neither spacing is particularly extreme. Secondary structure predictions by Sfold (60, 61) suggested that the UTR_pmyc1_*_tetO_* SD is likely to be in a single-stranded loop while the UTR*_sigA_* SD is likely to be partially base-paired (Fig. S2C and D), suggesting that there may be differences in SD accessibility for ribosome binding. Either the differences in SD start codon spacing or the differences in predicted secondary structure could potentially be responsible for the observed differences in apparent translation efficiency.

### Leaderless mRNAs may be transcribed less efficiently.

**FIG 4:**
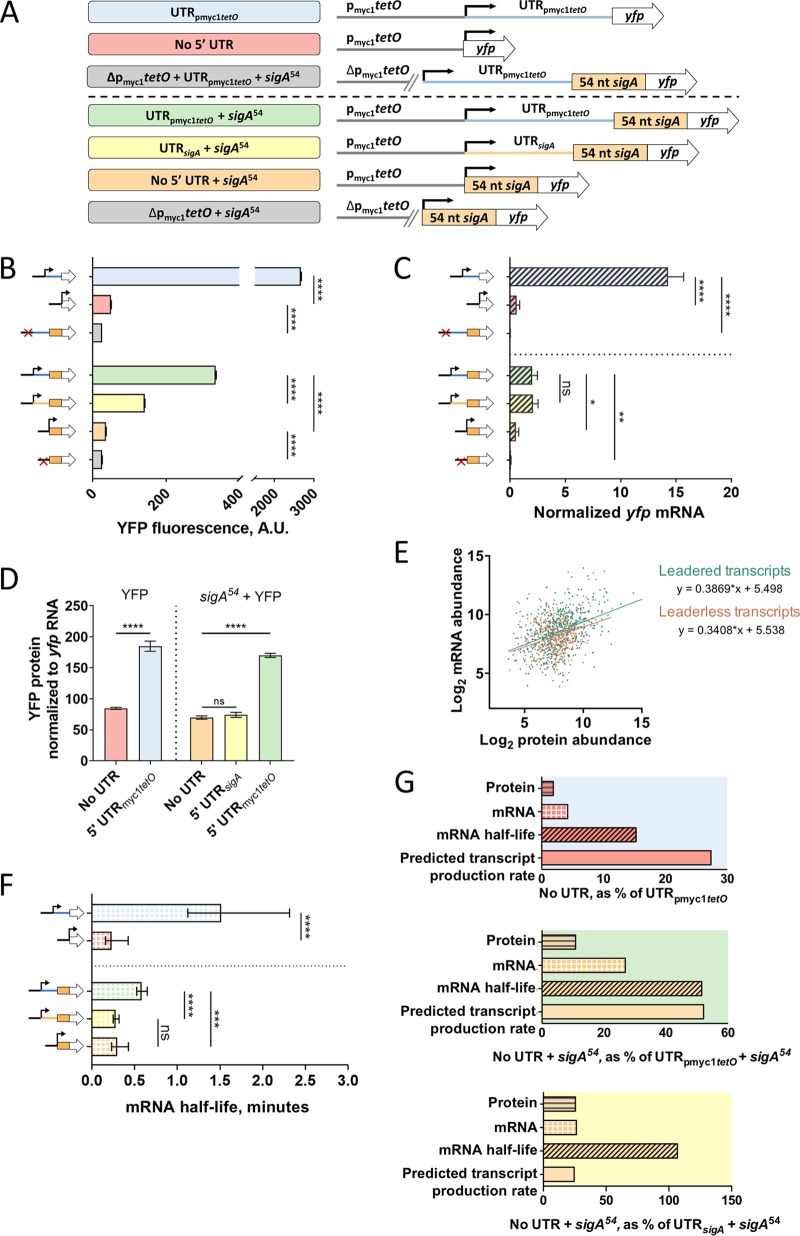
Leaderless transcripts have altered translation efficiencies, mRNA half-lives, and predicted transcript production rates compared to those of leadered controls. (A) Constructs transformed into M. smegmatis to compare leaderless versus leadered gene structures. (B) Median YFP fluorescence of strains bearing the constructs in panel A, determined by flow cytometry. Error bars denote 95% CI. Strains were compared by Kruskal-Wallis test followed by Dunn’s multiple-comparison test. (C) *yfp* mRNA abundance for strains bearing the indicated constructs, determined by qPCR and normalized to expression of endogenous *sigA*. Error bars denote standard deviation. Strains were compared by ANOVA with Tukey’s HSD. (D) Transcripts containing the p_myc1_*tetO*-associated 5′ UTR are translated more efficiently than leaderless transcripts or those containing the *sigA* 5′ UTR. (E) Published M. tuberculosis mRNA abundance (42) and protein abundance (62) levels for genes that have a single TSS and are leaderless or have 5′ UTRs of ≥15 nt. Protein and mRNA abundance were significantly correlated for both gene structures (*P* < 0.0001, Spearman’s ρ). Linear regression analysis revealed that the slopes were statistically indistinguishable (*P* = 0.44). (F) The half-lives of *yfp* mRNA produced from the indicated constructs were measured. Error bars denote 95% CI. Half-lives were compared using linear regression analysis (*n* = 3). (G) Protein abundance, mRNA abundance, mRNA half-life, and calculated transcript production rate for leaderless transcripts compared to transcripts with 5′ UTRs. Note that some data shown in Fig. 2 and 3 are reproduced here to facilitate comparisons. ****, *P* < 0.0001; ***, *P* < 0.001; ns, *P* > 0.05.

Leaderless transcripts are common in mycobacteria and were found to be associated with reduced protein abundance compared to that of leadered transcripts with near-consensus Shine-Dalgarno sites (43), suggesting that leaderless translation may be generally less efficient as was shown in E. coli (51–53). However, this hypothesis was not experimentally tested in mycobacteria. We therefore built two leaderless *yfp* reporters under the control of the p_myc1_*tetO* promoter, with and without the *sigA*^54^ N-terminal extension (Fig. 4A). When we compared YFP fluorescence between the leadered and leaderless reporters, we found that the leaderless fusions were substantially less fluorescent than those containing either 5′ UTR, regardless of the presence of *sigA*^54^ (Fig. 4B). The leaderless constructs also had reduced *yfp* mRNA levels compared to those of all of the leadered constructs (Fig. 4C). When comparing the leaderless constructs to the UTR_pmyc1_*_tetO_* construct, protein levels were decreased to a greater extent than mRNA levels, (Fig. 4D), suggesting that the leaderless mRNAs were indeed translated less efficiently than mRNAs bearing UTR_pmyc1_*_tetO_*. However, the difference in protein abundance from constructs without leaders and with UTR*_sigA_* could be largely explained by the difference in mRNA levels (Fig. 4D), suggesting that leaderless and UTR*_sigA_*-leadered mRNAs are translated with similar efficiencies.

To further evaluate the relationship between leader status and translation efficiency, we compared the relative abundances of proteins and mRNAs in M. tuberculosis using published quantitative proteomics data (62) and transcriptome sequencing (RNA-seq) data (42). For both leaderless transcripts and transcripts with 5′ UTRs ≥15 nt in length, mRNA abundance and protein abundance were significantly correlated (*P* < 0.0001, Spearman’s ρ) (Fig. 4E). We omitted transcripts with 1- to 14-nt UTRs because it is unknown if these behave more like leadered transcripts or more like leaderless transcripts with respect the mechanisms of translation initiation. Linear regression of these correlations revealed that they were statistically indistinguishable for leadered versus leaderless transcripts, consistent with the idea that variability in translation efficiency among mycobacterial genes is largely driven by factors other than the presence or absence of a leader.

We wondered if the reduced abundance of the leaderless *yfp* transcripts relative to the UTR_pmyc1_*_tetO_*-leadered transcripts was associated with reduced mRNA stability. Indeed, *yfp* half-lives for the UTR_pmyc1_*_tetO_* leadered transcripts were longer than their leaderless counterparts (Fig. 4F). In contrast, the leaderless transcripts had half-lives similar to the transcript bearing UTR*_sigA_* (Fig. 4F). Interestingly, leaderless transcripts with and without the *sigA*^54^ N-terminal extension had equivalent half-lives. Taken together, the data indicate that the destabilizing effect of *sigA*^54^ observed in Fig. 2F is dependent on the UTR_pmyc1_*_tetO_* present in those constructs.

The predicted *yfp* transcript production rates of the leaderless constructs were lower than those of their leadered counterparts (Fig. 4G). This is consistent with the idea that transcription-translation coupling can cause transcription rates to be altered as a function of translation efficiency (14). However, the UTR*_sigA_*-leadered transcript appeared to be translated with a similar efficiency as the leaderless constructs (Fig. 4D) and yet had a substantially higher transcript production rate.

### Translation efficiency is a poor predictor of mRNA half-life and transcript production rate.

The five constructs described above had identical promoters but produced strains that varied widely with respect to protein abundance, mRNA abundance, mRNA half-life, translation efficiency, and predicted transcript production rates. Given the reported impacts of translation efficiency on mRNA stability in bacteria (16–22), we wondered to what extent the differences in half-life among our constructs were explained by differences in translation efficiency. We defined translation efficiency as follows:

$$\text{translation efficiency}=\frac{\text{protein abundance}}{\text{[mRNA]}}$$

**FIG 5:**
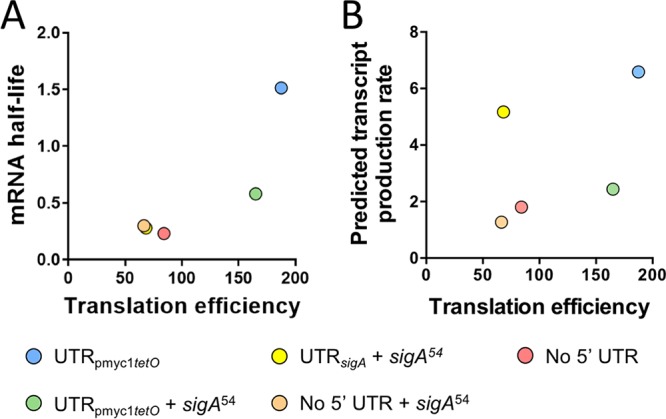
Translation efficiency is poorly correlated with mRNA half-life and predicted transcript production rate. Translation efficiency was defined as the ratio of protein abundance to mRNA abundance (arbitrary units). (A) Variability in mRNA half-life is largely not explained by variability in translation efficiency. (B) Variability in predicted transcript production rate is uncorrelated with translation efficiency.

However, the relationship between translation efficiency and mRNA half-life was weak (Fig. 5A), indicating that the variability in mRNA half-life was largely due to other factors. Translation rate has also been reported to affect transcription rate (14), but these two properties did not appear to be correlated in our constructs (Fig. 5B), suggesting that the differences in transcript production rate were not a consequence of differences in translation rate. Alternative interpretations of these and other analyses are discussed below.

## DISCUSSION

The *sigA* transcripts in both M. tuberculosis and M. smegmatis were reported to have relatively short half-lives (15, 63), and we hypothesized that this property was conferred in part by the 5′ UTR. We therefore sought to determine the impacts of the M. smegmatis *sigA* 5′ UTR on expression and mRNA stability. Compared to a 5′ UTR associated with high levels of protein expression and commonly used in mycobacterial expression vectors (57), the *sigA* UTR indeed conferred a shorter half-life as well as reduced translation efficiency (which could be the cause of the reduced half-life). However, the half-life of a *sigA*-leadered transcript was similar to that of a leaderless transcript. Insertion of part of the *sigA* coding sequence as an N-terminal translational fusion to our YFP reporter also caused a reduction in mRNA half-life. These findings suggest that the relative instability of the native *sigA* transcript is a product of multiple features, including the 5′ UTR and regions of the coding sequence. However, this effect was not observed for a leaderless version of the translational fusion, indicating the effect is context dependent.

Our mRNA abundance and half-life data allowed us to calculate predicted transcript production rates. These calculated transcript production rates reflect the combined contributions of transcription initiation, elongation, and termination (with initiation and termination likely being the largest contributors), and our methodology did not allow us to distinguish between these processes. It is important to note that these rates were not directly measured but rather inferred from direct measurements of mRNA abundance and half-life; other interpretations of the data are therefore possible, as described below. Interestingly, the *sigA* 5′ UTR appeared to increase transcript production rates compared to the p_myc1_*tetO*-associated UTR or leaderless transcripts. The promoter sequence upstream of the transcription start site (TSS) was identical for all constructs. The effect of the 5′ UTR on transcript production rate could therefore be mediated by at least three possible mechanisms. First, the sequence downstream of the TSS could affect RNA polymerase binding and therefore initiation as has been reported in E. coli (64). This is consistent with the finding that the E. coli RNA polymerase footprint extends 20 nt downstream of the TSS in both the open and closed initiation complexes (65) and advises caution when using TSS data to predict promoters. Second, the composition of the 5′ UTR could affect rates of premature termination and therefore rates of production of full-length transcript as has also been reported (19, 66). We also note that the qPCR primers that we used to measure transcript abundance and half-life anneal to the coding sequence, so we only quantify transcripts that extend at least 89 nt into the coding sequence. Transcripts that terminate before this point are not detected in our experimental setup. Third, elongation rates could vary among constructs due to presence or absence of pause sites (67) or DNA binding proteins that form roadblocks (68). Viewed broadly, this result highlights the complexity of bacterial transcription.

An alternative interpretation of these data is possible if one considers the idea that newly synthesized mRNAs may be degraded nearly instantaneously if not immediately engaged by the translation machinery. To our knowledge first proposed in detail in reference 58, this hypothesis invokes a population of “dark matter” mRNAs that are synthesized but decay so rapidly that they are not detected by abundance and half-life measurements. In this model, measured half-lives reflect primarily the decay of those transcripts that are quickly engaged by ribosomes and therefore long enough lived to contribute to steady-state abundance. The model implies that steady-state abundance is a function not only of transcription rate and measured half-life, as assumed in our transcript production rate calculations, but also of partitioning between the translated pool and the “dark matter” pool. If more mRNA is partitioned into the translated pool, greater steady-state abundance could be achieved without increased transcription rates and vice versa. Importantly, changes in partitioning between these pools need not correspond with predictable changes in measured mRNA half-life or protein levels. For example, transcripts containing the *sigA* 5′ UTR could conceivably engage ribosomes more quickly than transcripts containing the p_myc1_*tetO*-associated UTR and therefore enter the translated pool at higher rates yet be more susceptible to degradation during or after translation, resulting in shorter half-lives and thus similar steady-state mRNA abundance as we observed (Fig. 3).

The relative efficiency of leaderless versus leadered translation in mycobacteria has not been experimentally established. Proteomics data from M. tuberculosis suggested that proteins encoded on leaderless transcripts were less abundant than those encoded on leadered transcripts with evident SD sequences, but this difference appeared to be explained by differences in mRNA levels (43). A subsequently reported quantitative proteomics data set (62) allows for a more rigorous assessment of the relationship between mRNA abundance and protein abundance in M. tuberculosis. When comparing leaderless genes to leadered genes with a single TSS, we found that leaderless genes indeed on average had slightly but significantly lower levels of both mRNA and protein (Mann-Whitney tests for both, *P* < 0.01). However, the relationships between mRNA abundance and protein abundance did not differ for these two groups.

There are at least two ways to interpret protein/mRNA ratios. In Results and the figures, we refer to these ratios as a measure of translation efficiency on the grounds that they reflect the number of protein molecules produced per mRNA molecule. This interpretation rests upon the assumption that most mRNAs are stable enough to contribute to measurements of steady-state abundance. There are various reports of mutations or modifications that affect mRNA levels and protein levels differently (18, 19, 54, 66, 69), supporting the idea that translation efficiency can indeed be estimated by comparing protein levels to mRNA levels. Using this assumption and definition of translation efficiency, the M. tuberculosis data imply that there is no global difference in translation efficiency for leadered versus leaderless transcripts. Using the same assumptions, the small number of controlled comparisons that we report here support that idea; transcripts with the p_myc1_*tetO*-associated UTR were translated more efficiently than leaderless transcripts, but a transcript with the *sigA* UTR was translated with similar efficiency as its leaderless counterpart. Notably, the difference in translation efficiency between the two leadered transcripts might be attributable to differences in secondary structure rather than differences in favorability of the SD sequences.

A positive correlation between mRNA half-life and translation efficiency was reported for E. coli (70), consistent with the idea that translation may protect mRNAs from degradation. We did not observe such a correlation within our set of five transcripts, indicating that translation efficiency is not the primary driver of the variability in half-lives that we observed. However, a broad analysis of this relationship in mycobacteria is warranted.

The protein and mRNA abundance data must be interpreted differently if there is a “dark matter” mRNA pool that does not contribute to the mRNA abundance measurements. In this model, steady-state mRNA abundance is affected by translation efficiency as a consequence of partitioning between the translated pool and the “dark matter” pool. Translation efficiency would therefore affect steady-state mRNA abundance to a greater extent than that predicted by measured mRNA half-lives. Importantly, in this model, the relationship between protein abundance and mRNA abundance is not a reliable indicator of translation efficiency. In M. tuberculosis, lower average levels of both mRNA and protein from leaderless genes could therefore be a consequence of slower engagement of ribosomes and greater partitioning of mRNAs into the undetected “dark matter” pool.

It is prudent to note the assumptions underlying our definition of mRNA half-life. We assume that following transcription block by rifampin, the initial rapid decrease in *yfp* transcript abundance that we observe (see Fig. S3 in the supplemental material) indeed reflects the degradation rate for most *yfp* transcripts produced in an unperturbed cell. This assumption could be invalid if rifampin immediately alters mRNA degradation rates. If there are populations of “dark matter” mRNAs, the measured half-lives presumably reflect only the decay rate of those mRNAs that are engaged by ribosomes. We also note that, for most constructs, we observed a second, slow phase of mRNA decay (see Fig. S3 and Materials and Methods). This could reflect changes in mRNA decay in response to rifampin or reflect the presence of a second pool of transcripts with an inherently slower decay rate. As we could not distinguish between these possibilities, and the slow-decaying pool appeared to comprise at most 10% of the total, we did not further analyze it in this study. However, we cannot exclude the possibility that it is real and physiologically relevant.

## MATERIALS AND METHODS

### Strains and culture conditions.

All experiments were done using a Mycobacterium smegmatis Δ*MSMEG_2952* strain (71), which is less prone to aggregation (clumping) than its parent strain mc^2^155 and therefore permits higher confidence measurements by flow cytometry. This strain and its derivatives (Table 1) were grown in Middlebrook 7H9 medium with albumin-dextrose-catalase (ADC) supplementation (final concentrations, 5 g/liter bovine serum albumin fraction V, 2 g/liter dextrose, 0.85 g/liter NaCl, and 3 mg/liter catalase), 0.2% glycerol, and 0.05% Tween 80. Cultures were shaken at 200 rpm and 37°C to an optical density at 600 nm (OD_600_) of ∼0.8 at the time of harvest.

**TABLE 1:** Strains and plasmids

Plasmid	Strain (reference)	Characteristics
	Δ*MSMEG_2952* strain (71)	mc^2^155 *MSMEG_2952*::Hyg*^r^*
pSS303	SS-M_0486	p_myc1_*tetO* promoter + p_myc1_ 5′ UTR + *yfp*-6×His
pSS309	SS-M_0489	p_myc1_*tetO* promoter + *sigA* 5′ UTR + first 54 nt of *sigA* + *yfp*-6×His
pSS310	SS-M_0493	p_myc1_*tetO* promoter + no 5′ UTR + *yfp*-6×His
pSS314	SS-M_0497	p_myc1_*tetO* promoter with a deletion of nt −53 through −1 + *yfp*-6×His
pSS316	SS-M_0521	p_myc1_*tetO* promoter + Δ1GTG *sigA* + first 54 nt of *sigA* + *yfp*-6×His
pSS335	SS-M_0524	p_myc1_*tetO* promoter + Δ2GTG *sigA* + first 54 nt of *sigA* + *yfp*-6×His
pSS359	SS-M_0623	p_myc1_*tetO* promoter + p_myc1_ 5′ UTR + first 54 nt of *sigA* + *yfp*-6×His
pSS360	SS-M_0626	p_myc1_*tetO* promoter + no 5′ UTR + first 54 nt of *sigA* + *yfp*-6×His
pSS365	SS-M_0629	p_myc1_*tetO* promoter with a deletion of nt −53 through −1 + first 54 nt of *sigA* + *yfp*-6×His
pSS384	SS-M_0636	p_myc1_*tetO* promoter with a deletion of nt −53 through −1 + *sigA* 5′ UTR + first 54 nt of *sigA* + *yfp*-6×His
pSS385	SS-M_0639	p_myc1_*tetO* promoter with a deletion of nt −53 through −1 + p_myc1_ 5′ UTR + first 54 nt of *sigA* + *yfp*-6×His

### Plasmid construction.

Plasmid pSS303 was built on a backbone derived from pGH1000A (72) by inserting a *yfp* cassette containing the gene sequence of a YFP reporter (sfYFP, obtained from Ivy Fitzgerald and Benjamin Glick) with a 6×His tag at the C terminus (complete amino acid sequence, MASDSTESLFTGVVPILVELDGDVNGHKFSVRGEGEGDATNGKLTLKLICTTGKLPVPWPTLVTTLGYGVQCFARYPDHMKQHDFFKSAMPEGYVQERTITFKDDGTYKTRAEVKFEGDTLVNRIELKGIDFKEDGNILGHKLEYNFNSHNVYITADKQKNGIKANFKIRHNVEDGGVQLADHYQQNTPIGDGPVLLPDNHYLSYQSKLSKDPNEKRDHMVLLEFVTAAGITHGSSGSSGCHHHHHH). Two synthetic transcriptional terminators were inserted flanking the cassette as follows: *tsynA* (73) upstream and *ttsbiB* (74) downstream. Transcription was initiated by the p_myc1_*tetO* promoter, which was constitutively active in our strains due to the absence of the corresponding *tet* repressor (57). All constructs (pSS303 and derivatives noted in Table 1) were built using NEBuilder HiFi DNA assembly master mix (catalog number E2621). Each assembled plasmid was integrated in M. smegmatis Δ*MSMEG_2952* (71) at the Giles phage site and selected with 200 μg/ml hygromycin.

### Cell fixation and flow cytometry.

Several 1.5-ml aliquots of M. smegmatis cultures were pelleted, resuspended in 500 μl 2% paraformaldehyde in phosphate-buffered saline (PBS), and incubated at room temperature for 30 min. Cells were rinsed twice using 900 μl PBS + 0.1% Tween 20 and resuspended to a calculated OD_600_ of 15. Prior to flow cytometry analysis, cells were filtered using an 18-gauge 5-μm filter needle and diluted with Middlebrook 7H9 to an OD_600_ of 0.015. YFP fluorescence intensity was measured per manufacturer’s instructions using a BD Accuri C6 flow cytometer collecting 100,000 events per sample (Fig. 1B and C) or a BD LSR II flow cytometer collecting 50,000 events per sample (Fig. 2B, 3B, and 4B) using appropriate controls and thresholds. FlowJo v10.6 was used to draw tight forward scatter and side scatter gates to limit analysis to similarly sized cells, and GraphPad Prism 8 was used for statistical analysis.

### RNA extraction and determination of mRNA abundance and stability.

RNA extraction, measurement of mRNA abundance, and mRNA stability analyses from M. smegmatis cultures were conducted in biological triplicates as described in reference 15. Briefly, mRNA abundance was measured by quantitative PCR (qPCR) using iTaq SYBR green (Bio-Rad) on an Applied Biosystems 7500 with 400 pg of cDNA and 0.25 μM each primer in 10-μl reaction mixtures. Cycle parameters were 95°C for 15 s and 61°C for 60 s. Primers used to determine mRNA abundance are listed in Table 2.

**TABLE 2:** Primers for qPCR

Primer name (reference)	Gene	Directionality	Sequence 5′ → 3′
JR273 (75)	*sigA* (*MSMEG_2758*)	Forward	GACTACACCAAGGGCTACAAG
JR274 (75)	*sigA* (*MSMEG_2758*)	Reverse	TTGATCACCTCGACCATGTG
SSS833	*yfp*	Forward	GATAGCACTGAGAGCCTGTT
SSS834	*yfp*	Reverse	CTGAACTTGTGGCCGTTTAC

For mRNA stability analysis, 5-ml M. smegmatis cultures were treated with rifampin at a final concentration of 150 μg/ml to halt transcription and snap-frozen in liquid nitrogen after 0, 1, 2, or 4 min. Abundance over time was determined for *sigA* and *yfp* using qPCR and used to estimate mRNA half-lives essentially as in reference 15. For each sample, the negative of the threshold cycle (*C_T_*) represents transcript abundance on a log_2_ scale. For each strain and gene, linear regression was performed on a plot of −*C_T_* versus time. Half-life was defined as −1/slope. *sigA* half-lives were equivalent in all strains and not shown. As we have observed for many other genes in mycobacteria, plotting log_2_ abundance over time produced a biphasic decay curve consistent with a period of faster exponential decay, followed by a period of much slower exponential decay (see Fig. S3A in the supplemental material). Similar biphasic decay curves have been reported by others for some E. coli genes (76–79). The initial rapid decay phase reflects the rate of decay for at least 90% of the *yfp* RNA present in our samples. We therefore used only this initial phase for mRNA half-life calculations (0, 1, and 2 min for strain SS-M_0489 and 0 and 1 min for strains SS-M_0493 and SS-M_0626) (see Fig. S3B). The slower decay phase could reflect the presence of minority transcript species that inherently decay more slowly or could reflect perturbation of cellular physiology due to rifampin.

### Calculation of transcript production rates.

The rate of transcript production was estimated as described in reference 59. Briefly, transcript production rate (*V_t_*) is described as follows:

$$V_{t}=k\cdot \left[\text{mRNA}\right]+\text{μ}\cdot [\text{mRNA}]$$

Where [mRNA] is a given transcript’s concentration, μ is the growth rate of the cells (ln_2_/doubling time), and *k* is the degradation rate constant (ln_2_/half-life). Note that because [mRNA] is derived from our qPCR data and is therefore a relative value rather than absolute value, the calculated transcript production rate is also a relative rather than absolute value.

### Protein extraction and BCA assay.

M. smegmatis cells were pelleted; rinsed three times with Middlebrook 7H9, 0.2% glycerol, and 0.05% Tween 80 at 4°C; resuspended in PBS + 2% SDS + protease inhibitor cocktail (VWR; catalog number 97063-972); and transferred to 2-ml disruption tubes (OPS Diagnostics; 100-μm zirconium lysing matrix, molecular grade). Cultures were lysed using a FastPrep-24 5G instrument (MP Biomedical) using four cycles of 6.5 m/s for 30 s with 1 min on ice between cycles. Samples were clarified by centrifugation at 21,130 × *g* at 4°C for 10 min, and the supernatant containing protein was recovered and stored at –20°C. Protein concentrations were calculated using the Pierce BCA protein assay (Thermo Scientific; catalog number 23225) according to the manufacturer’s instructions.

### Western blotting.

Protein was normalized to the indicated masses in a final volume of 9 μl combined with 4 μl of 4× protein loading dye (200 mM Tris-HCl [pH 6.8], 400 mM dithiothreitol [DTT], 8% SDS, 0.4% bromophenol blue, 40% glycerol) and heated to 95°C for 10 min. Using gradient gels (4 to 15% Mini-Protean TGX precast protein gels; Bio-Rad; catalog number 4561086), the samples were electrophoresed for 60 min at 140 V and then transferred to a polyvinylidene difluoride (PVDF) membrane. The membrane was incubated in blocking solution (PBS plus 5% nonfat milk) for 30 min and washed once for 5 min using washing buffer (PBS 1× buffer plus 0.1% Tween 20). The membrane was probed with 1 μg/ml His tag antibody (polyclonal antibody, rabbit; GenScript; catalog number A00174) in blocking solution for 60 min at room temperature. The membrane was then rinsed twice with wash buffer and once with 1× PBS and incubated with anti-rabbit IgG–peroxidase (Sigma-Aldrich; catalog number A4914), 1:30,000 in blocking solution, for 60 min at room temperature. The membrane was rinsed as previously described and incubated with horseradish peroxidase (HRP) substrate (Radiance Q; Azure Biosystems; catalog number AC2101) as recommended by the manufacturer. Imaging was done using an Azure C200 imaging system (Azure Biosystems).

### Software.

GraphPad Prism was used for all linear regressions and comparisons (GraphPad Software, La Jolla, CA). The Srna program within Sfold was used for RNA secondary structure predictions (60, 61).

## Supplementary Material

Supplemental file 1

Supplemental file 2
