# Supplementary material for: The Impact of Leadered and Leaderless Gene Structures on Translation Efficiency, Transcript Stability, and Predicted Transcription Rates in Mycobacterium smegmatis
Source: J Bacteriol. 2020 Apr 9;202(9):e00746-19. doi: 10.1128/JB.00746-19 (PMC7148126; doi:10.1128/JB.00746-19)
Supplement: Supplemental file 1 [file JB.00746-19-s0002.pdf]

SUPPLEMENTAL TABLE

Supplemental Table 1. *M. smegmatis* and *M. tuberculosis* 5' UTRs between 6 and 300 nt in length.

SUPPLEMENTAL FIGURES

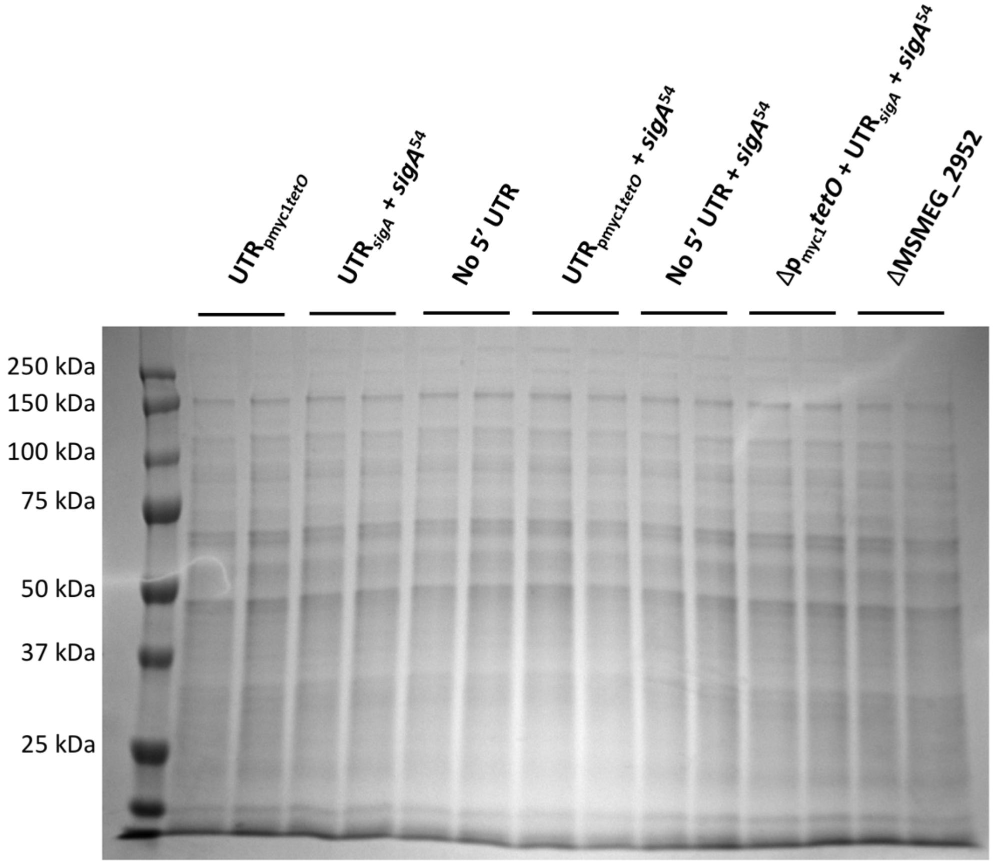

Figure S1. Expression of YFP constructs does not appear to globally affect protein levels in *M. smegmatis*. Coomassie stained gel loaded with duplicate lysates from strains expressing the indicated YFP constructs (first six strains) or from the parental strain into which the YFP constructs were transformed (last strain). YFP is approximately 27 kDa and does not appear to be expressed at high enough levels to be visible by Coomassie staining.

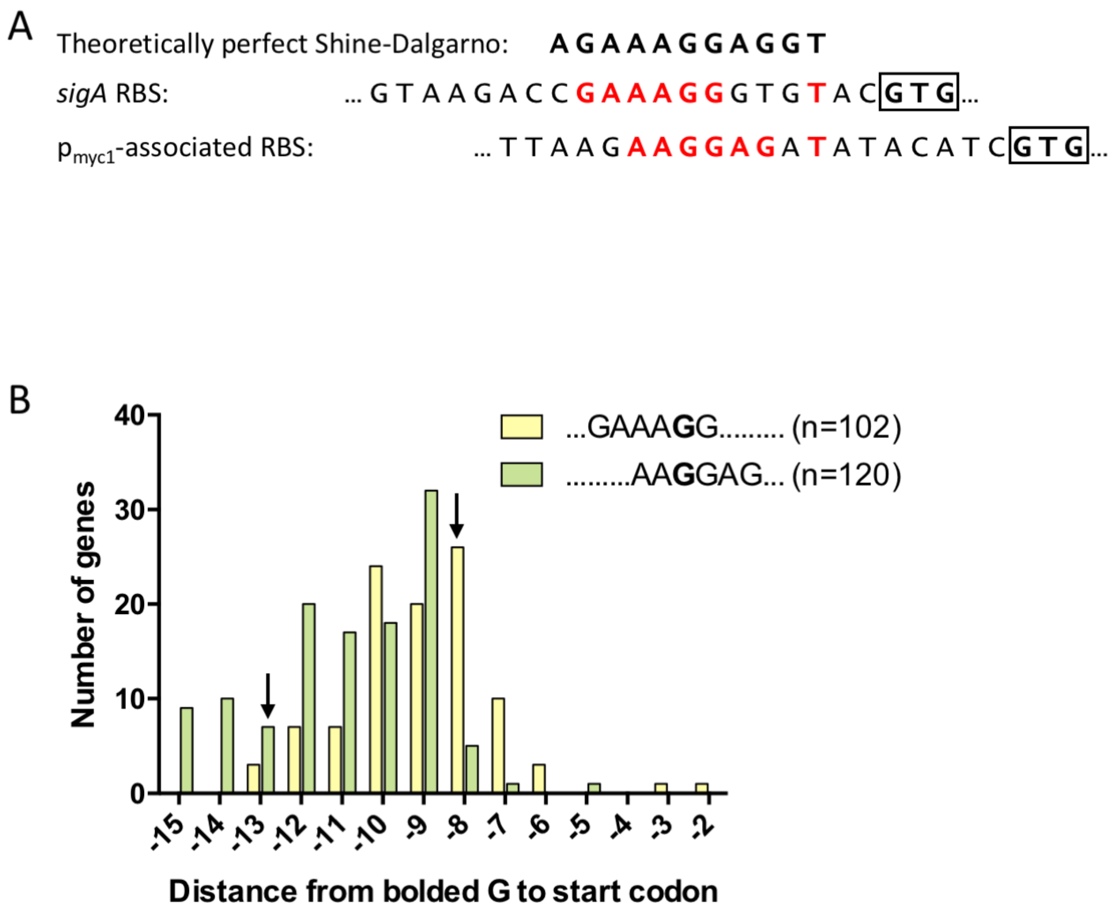

Figure S2. Comparison of Shine-Dalgarno (SD) sequences and predicted secondary structures for the *sigA* 5' UTR and the p_{myc1}*tetO*-associated 5' UTR. A: The *sigA* and p_{myc1}*tetO*-associated RBSs are shown aligned to the reverse complement of the 3' end of the *M. smegmatis* 16S rRNA. Positions that match this theoretically perfect SD sequence are highlighted in red. Start codons are bolded and boxed. **B:** Distributions of SD-start codon spacings for all genes that have the indicated SD sequences in the *M. smegmatis* genome. Yellow indicates the *sigA* SD sequence and green indicates the p_{myc1}*tetO*-associated SD sequence. Black arrows indicate the SD-start codon spacings for the *sigA* and p_{myc1}*tetO*-associated SD sequences. **C-D:** Ensemble centroid predictions (Sfold, [60]) for secondary structures formed by the $p_{myc1tetO}$-associated (C) and *sigA* (D) 5' UTRs plus the first 15 nt of the *sigA* coding sequence. The predicted core SD sequences are highlighted in red. Start codons are highlighted in gray. The structures of the RBS regions were predicted to be the same when folding was performed using only the UTRs and start codons or using the UTRs and 54 nt of the *sigA* coding sequence.

C

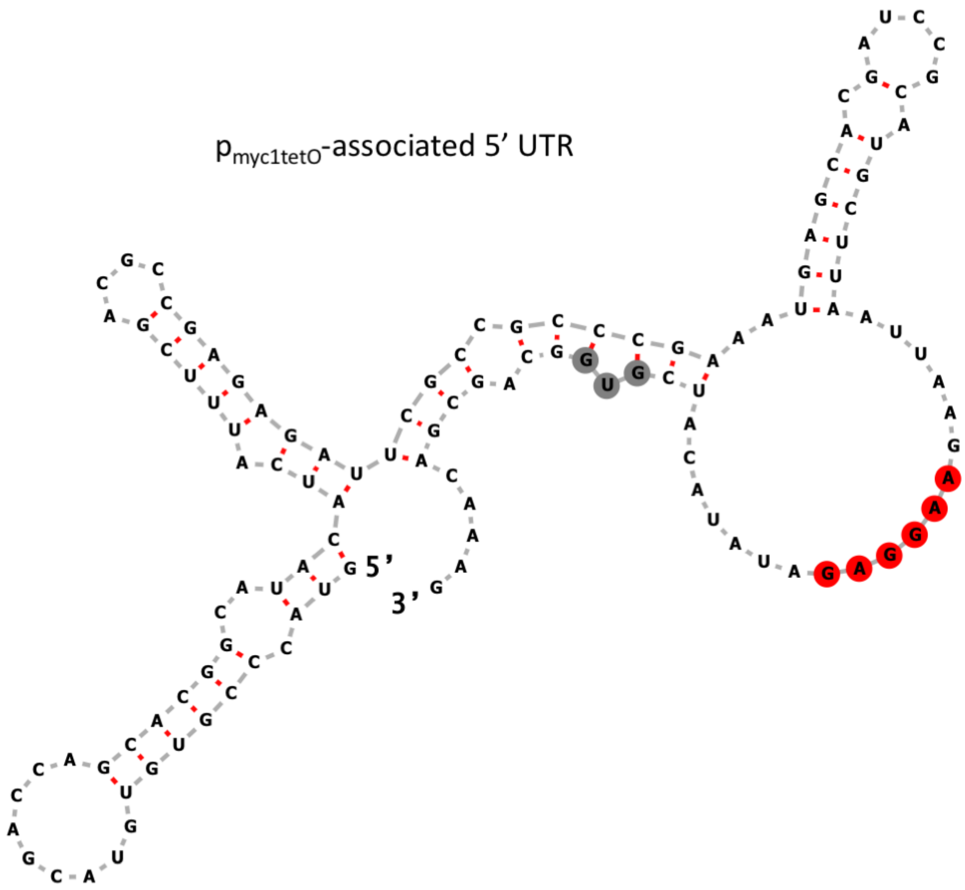

D

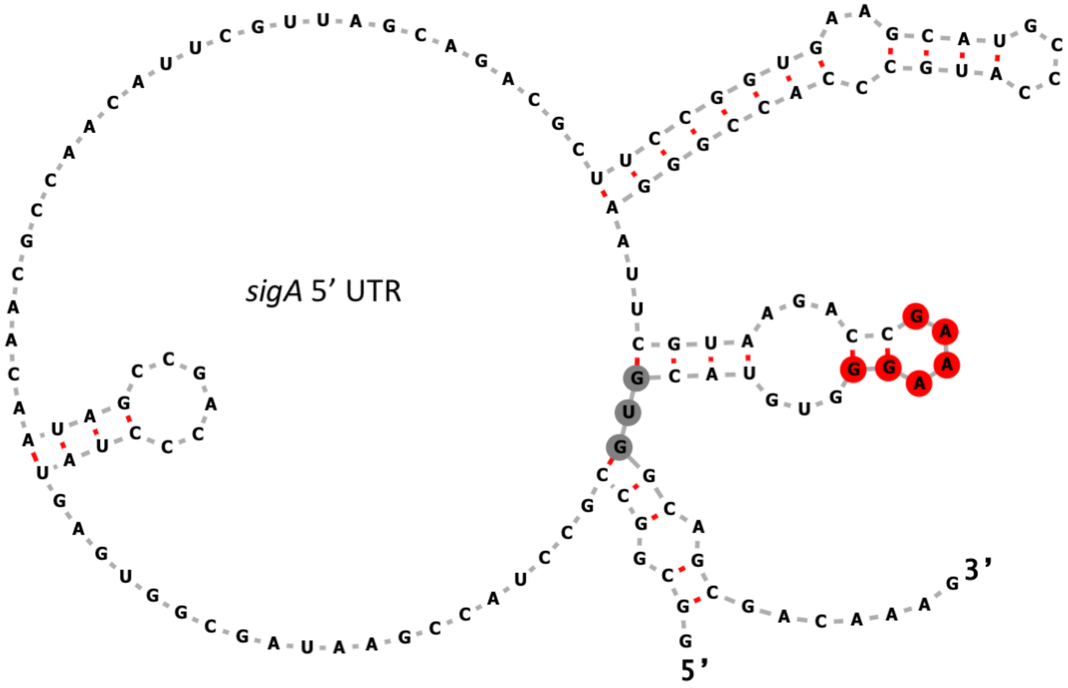

A

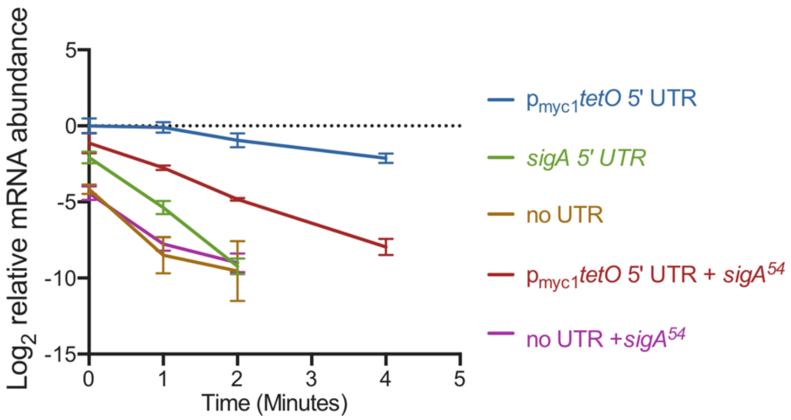

B

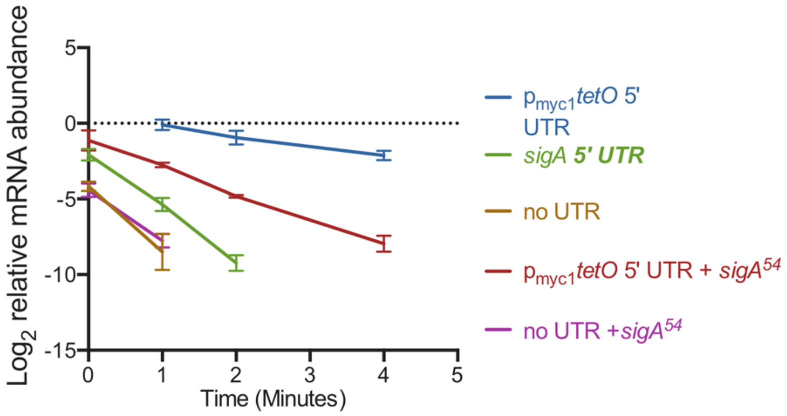

Figure S3. mRNA decay curves used to calculate the half-lives reported in the main text. A. Quantitative PCR was used to measure *yfp* abundance following treatment of *M. smegmatis* cultures with 150 µg/ml rifampicin at time zero. The values displayed are normalized to the time zero abundance for the $p_{myc1}^{tetO5'UTR}$ construct and log2 transformed. A single exponential decay is expected to produce a straight line for log2 transformed values. In the main text we discuss possible reasons for the biphasic curves produced by some constructs. B. The timepoints used for calculating half-lives. The steepest portion of each curve was used, which in most cases was the earlier timepoints. As further discussed in the main text, we predicted that the earlier, steeper decay rates were most likely to reflect the decay rates of the majority of the RNA molecules in cells not perturbed by rifampicin.
